# An Evolutionarily Conserved Synthetic Lethal Interaction Network Identifies FEN1 as a Broad-Spectrum Target for Anticancer Therapeutic Development

**DOI:** 10.1371/journal.pgen.1003254

**Published:** 2013-01-31

**Authors:** Derek M. van Pel, Irene J. Barrett, Yoko Shimizu, Babu V. Sajesh, Brent J. Guppy, Tom Pfeifer, Kirk J. McManus, Philip Hieter

**Affiliations:** 1 Michael Smith Laboratories, University of British Columbia, Vancouver, Canada; 2 Department of Biochemistry and Molecular Biology, University of British Columbia, Vancouver, Canada; 3 Department of Screening, Centre for Drug Research and Development, Vancouver, Canada; 4 Manitoba Institute of Cell Biology, Department of Biochemistry and Medical Genetics, University of Manitoba, Winnipeg, Canada; University of Washington, United States of America

## Abstract

Harnessing genetic differences between cancerous and noncancerous cells offers a strategy for the development of new therapies. Extrapolating from yeast genetic interaction data, we used cultured human cells and siRNA to construct and evaluate a synthetic lethal interaction network comprised of chromosome instability (CIN) genes that are frequently mutated in colorectal cancer. A small number of genes in this network were found to have synthetic lethal interactions with a large number of cancer CIN genes; these genes are thus attractive targets for anticancer therapeutic development. The protein product of one highly connected gene, the flap endonuclease *FEN1*, was used as a target for small-molecule inhibitor screening using a newly developed fluorescence-based assay for enzyme activity. Thirteen initial hits identified through *in vitro* biochemical screening were tested in cells, and it was found that two compounds could selectively inhibit the proliferation of cultured cancer cells carrying inactivating mutations in *CDC4*, a gene frequently mutated in a variety of cancers. Inhibition of flap endonuclease activity was also found to recapitulate a genetic interaction between *FEN1* and *MRE11A*, another gene frequently mutated in colorectal cancers, and to lead to increased endogenous DNA damage. These chemical-genetic interactions in mammalian cells validate evolutionarily conserved synthetic lethal interactions and demonstrate that a cross-species candidate gene approach is successful in identifying small-molecule inhibitors that prove effective in a cell-based cancer model.

## Author Summary

Anticancer therapeutic discovery is a major challenge in cancer research. Because cancer is a disease caused by somatic genetic mutations, the search for anticancer therapeutics is often driven by the ability to exploit genetic differences specific to tumor cells. Recently, cancer therapeutic development has sought to exploit synthetic lethality, a situation in which the combination of two independently viable mutations results in lethality. If a compound can be found to selectively kill a specific genotype via inhibition of a specific gene product, this is known as a chemical-genetic interaction, and it mimics a synthetic lethal genetic interaction. The ideal therapeutic would be broad spectrum, that is, active against multiple cancer genotypes within a tumor type and/or across a variety of cancers. We have developed an approach, taking advantage of the evolutionary conservation of synthetic lethal interactions, to identify “second-site” targets in cancer: genes whose chemical inhibition leads to selective killing of tumor cells across a broad spectrum of cancer genotypes. We identified small-molecule inhibitors of one such target, FEN1, and showed that these compounds were able to selectively kill human cells carrying cancer-relevant mutations. This approach will facilitate the development of anticancer therapeutics active against a variety of cancer genotypes.

## Introduction

Cancerous cells carry somatic mutations that genotypically distinguish them from surrounding noncancerous cells, and this provides an opportunity that can be exploited for therapeutic development. One strategy for the specific targeting of cancer genotypes relative to nonmutated somatic cells is to exploit synthetic lethal interactions [1]. For example, breast cancer cells with mutations in *BRCA1* or *BRCA2* are extremely susceptible to knockdown or chemical inhibition of *PARP1*, which encodes poly(ADP)ribose polymerase (PARP) [2], [3].

While exploiting synthetic lethality has the potential to be an effective approach to treating tumors, a major challenge is the identification of clinically relevant small-molecule inhibitors. One approach, pioneered by the National Cancer Institute, is to screen many thousands of unknown potential therapeutics on cancer cell lines [4]. Compounds generate a “fingerprint” of activity against certain cell lines, which can then be deconvolved, usually by mutation sequencing, to yield novel gene-drug interactions, in a so-called “bottom-up” approach. Alternatively, a “top-down” approach applies compounds of known target or mode of action to known genotypes, again to identify new gene-drug interactions. Recently, two groups used such an approach to screen more than 100 compounds against hundreds of cancer cell lines whose mutational status was known [5], [6], observing that gene-drug interactions tended to be more significant for targeted therapies, such as compounds targeting the *BCR-ABL* fusion protein, than for generally cytotoxic drugs, such as DNA damaging agents or antimitotics [6]. Thus, screening for compounds targeting a specific genetic lesion is preferable to developing new cytotoxic agents. Such targeted compounds can then be deployed as first-line anticancer therapeutics either singly or in a combination regime that would lessen the likelihood of drug-resistant clones developing within the tumor cell population [7], [8].

Many different cancer mutations lead to a limited repertoire of cancer phenotypes, such as chromosome instability, checkpoint dysfunction, and hyperplasia [9]. It is possible to identify a gene target that results in synthetic lethality with a large number of unlinked gene mutations by screening for targets that result in synthetic lethality with a common tumor phenotype. For example, chromosome instability (CIN), an increase in the rate of gain or loss of whole or parts of chromosomes, is observed in the form of aneuploidy in more than 90% of solid tumors and over 75% of blood cancers [10]. As the maintenance of genomic stability is an essential cellular process, CIN represents a phenotype that could potentially be leveraged towards selective killing of cancerous cells relative to normal cells. A gene that is synthetic lethal with a large number of cancer-related CIN genes would be an attractive therapeutic target in a large fraction of tumors.

Genetically tractable model organisms, such as the budding yeast *Saccharomyces cerevisiae*, facilitate the identification of human CIN genes, via identification and sequencing of their human orthologs. For example, identification of yeast CIN genes [11]) led to the sequencing of the human homologs of 200 yeast CIN genes in human colorectal cancers, and it was discovered that human homologs of the yeast CIN genes *SMC1*, *SCC2*, *BUB1*, *PDS1*, *MRE11*, and *CDC4* collectively account for approximately 25% of the mutational spectrum of colorectal cancer [12]–[15]. Thus, if a common synthetic lethal interacting partner could be identified for all of these genes, and a highly potent and specific inhibitor of its activity could be developed, inhibition of this target would offer a potentially broad means of targeting CIN cancers. In yeast, technologies exist to screen for genome-wide synthetic lethal interactions with relative ease [16], and identification of the synthetic lethal interaction network of the yeast orthologs of cancer-mutated genes has in previous cases revealed a small number of “hub” genes having synthetic lethal interactions with many yeast cancer-orthologs [17]. Previous studies have found a high degree of conservation between yeast and metazoan genetic interactions [18], [19], suggesting hub gene identification based on a yeast CIN gene synthetic lethal interaction network should yield broad-spectrum, second-site target genes applicable to human cancers.

Here we present and validate a cross-species candidate-based approach to the identification of anticancer targets and the discovery of anticancer therapeutics. We show that a genetic interaction network comprised of colorectal cancer CIN genes is largely conserved between *S. cerevisiae* and a human cancer cell line. We develop an *in vitro* assay for the activity of the protein encoded by one such highly connected gene, *FEN1*, and use this assay to screen for small-molecule inhibitors. Finally, we show that flap endonuclease inhibitors recapitulate conserved genetic interactions. These data demonstrate the effectiveness of a cross-species synthetic lethal approach to the discovery of potential anticancer therapeutics.

## Results

### A cross-species approach reveals conserved genetic interaction partners of cancer genes

**Figure 1:**
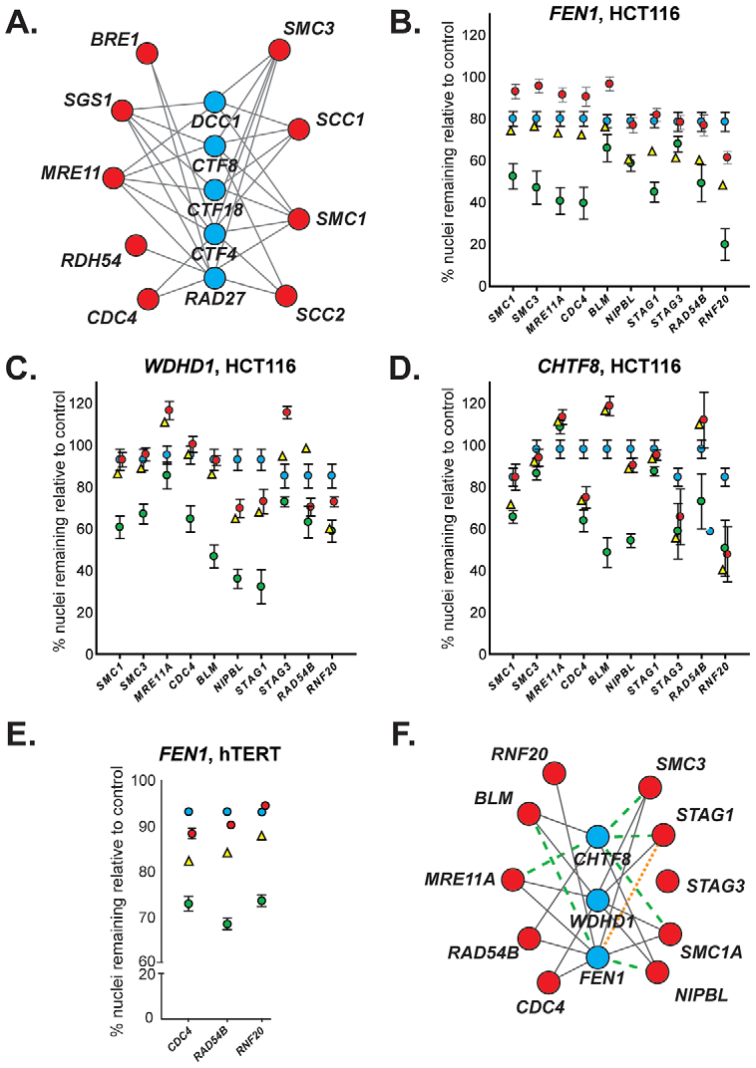
Evolutionary conservation of synthetic lethal interactions in HCT116 cells. (A) A yeast cancer-ortholog synthetic lethal network. Lines indicate synthetic lethal genetic interactions. Yeast genes and human orthologs are presented in Table 1. Red circles represent *S. cerevisiae* orthologs of genes mutated in cancer; blue circles indicate common interacting partners, which are referred to as “central” genes. (B–D) Representative data depicting mean percentage of remaining HCT116 cells (± SEM) treated with pooled siRNAs targeting central genes (top) and cancer genes (along *x*-axis) relative to GAPDH-silenced controls. Blue circles, siRNA targeting central gene alone. Red circles, siRNA targeting cancer gene alone. Yellow triangles, predicted viability of double siRNA treatment. Green circles, observed viability of double siRNA treatment. For raw data, please refer to . GAPDH siRNA does not significantly reduce viability relative to a non-silencing siRNA (Figure S2A). (E) Representative data depicting mean percentage viability of hTERT cells (± SEM) treated with pooled siRNAs targeting FEN1 and cancer genes (along *x*-axis) relative to GAPDH-silenced controls. Symbols are as in B. (F) Mammalian genetic interaction network. Solid grey line, interaction observed in both *S. cerevisiae* and HCT116 cells; green dashed line, interaction observed only in *S. cerevisiae*; orange dotted line, interaction observed only in HCT116 cells.

The human genes *SMC1*, *SMC3*, *NIPBL*, *STAG3*, *RNF20*, *FBXW7/CDC4*, *MRE11A*, *RAD54B* and *BLM* have been found to be mutated in colorectal cancer, and together account for approximately 25% of the CIN mutational spectrum of this disease [13]–[15], [20]–[22]. Protein BLAST was used to identify the budding yeast orthologs of these human genes (Table 1) and we constructed a synthetic lethal interaction network (Figure 1A), using literature and publicly available genetic interaction data (BioGrid and the Saccharomyces Genome Database) [18], [23].

**Table 1:** Yeast and human gene orthologs.^A^

Yeast gene	Human ortholog(s)	% identity	% similarity	BLASTP expectation
*BRE1*	*RNF20*	21%	42%	4×10^−26^
*CDC4*	*CDC4* (*FBXW7*)	29%	51%	4×10^−63^
*CTF18*	*CHTF18*	24%	42%	8×10^−36^
*CTF4*	*WDHD1* (*AND1*)	21%	36%	9×10^−18^
*CTF8*	*CHTF8*	21%	37%	6×10^−19^ B
*DCC1*	*DSCC1*	24%	39%	10^−10^
*MRE11*	*MRE11A*	41%	59%	5×10^−128^
*RAD27*	*FEN1*	60%	76%	2×10^−137^
*RDH54*	*RAD54B*	37%	52%	3×10^−134^
***SCC1*** ** (** ***MCD1*** **)**	***RAD21***	**35%**	**54%**	**2×10^−7^**
***SCC2***	***NIPBL***	**20%**	**39%**	**10^−18^**
***SCC3 (IRR1)***	***STAG1*** **, ** ***STAG2*** **, ** ***STAG3***	**26%**	**46%**	**10^−20^**
*SGS1*	*BLM*, *WRN*	39%	60%	4×10^−121^
***SMC1***	***SMC1A***	**30%**	**54%**	**10^−163^**
***SMC3***	***SMC3***	**32%**	**55%**	**0**

A Names indicated are the names used in this work. Names in parentheses indicate common alternative gene names. Members of the cohesin complex (and *SCC2/NIPBL*, a cohesin loader) are indicated in boldface type.

B Identified with DELTA BLAST algorithm.

To investigate the conservation of this network between yeast and a human cell line, we used siRNA-mediated knockdown of potential synthetic lethal gene pairs in the cell line HCT116. Knockdown efficiencies were evaluated by Western blots (Figure S1A). All pair-wise combinations between the three “central” synthetic lethal partner genes, *WDHD1*, *FEN1*, and *CHTF8*, and the ten outer cancer-mutated CIN genes were evaluated for synthetic lethality (Figure 1B, 1C, 1D). (*CHTF8* was selected as a representative of the alternative RFC^CHTF18^, comprised of Dcc1, Ctf8, and Ctf18 in *S. cerevisiae*).

Of the 30 possible synthetic lethal interactions among the genes tested, 22 have been reported in yeast [18], [23]. We found 16 of the predicted interactions (73%) were conserved between yeast and human cells, and 6 predicted interactions did not appear conserved in our assay (27%). Furthermore, one interaction, between *FEN1* and *STAG1*, was not predicted based on yeast data; however, we detected a genetic interaction between these genes (Figure 1F and Table S1). No interactions were observed with *STAG3*, which functions primarily in human meiosis [24]. As in yeast, all three central genes – *WDHD1*, *FEN1*, and *CHTF8* – were highly connected to sister chromatid cohesion genes (e.g. cohesin and/or cohesin loaders) (Figure 1F).

As *FEN1* encodes an enzyme, whereas *WDHD1* and *CHTF8* do not; it may be amenable to biochemical inhibitor screening. Thus, we sought to further validate genetic interactions between *FEN1* and other genes in the network. To ensure that these observed interactions were not cell line-dependent, we attempted to recapitulate interactions between *FEN1* and each of *CDC4*, *RAD54B*, and *RNF20* in the karyotypically stable, immortalized fibroblast cell line hTERT. As in HCT116 cells, genetic interactions were observed following knockdown of all three gene pairs (Figure 1E, Table S2, Figure S1C). We found that individual siRNAs could recapitulate the genetic interactions observed with the pooled siRNAs (Table S3). These data validate a subset of genetic interactions identified in the HCT116 cells and thus confirm FEN1 as a strong candidate therapeutic target.

### Developing a high-throughput *in vitro* assay for FEN1 activity

**Figure 2:**
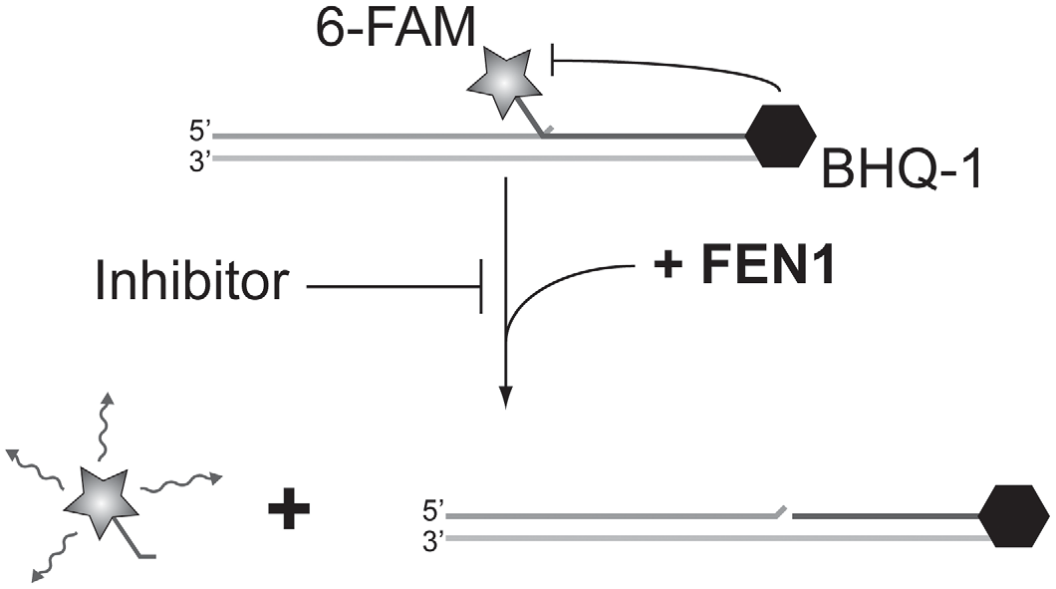
Screening for FEN1 inhibitors *in vitro*. Schematic representation of the fluorescence-based assay employed to identify FEN1 inhibitors. In the absence of inhibitor, FEN1 cleaves the 5′ flap to which the 6-FAM fluorophore is attached, allowing it to diffuse away from the BHQ-1 quencher and fluoresce. Activity is read as increasing fluorescence over time.

**Figure 3:**
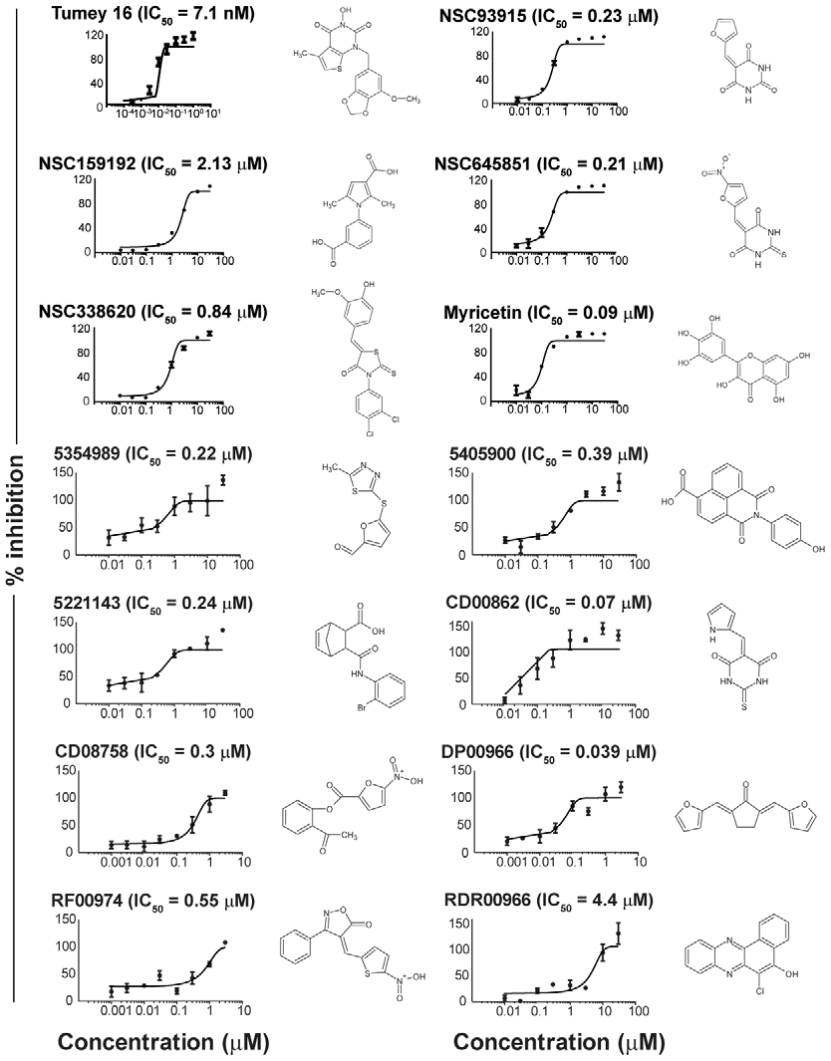
IC_50_ curves of flap endonuclease inhibitors. FEN1 assays were carried out as described in Materials and Methods. Compound names are indicated above each graph, and structures are given to the right of each graph. Tumey 16 (top-left panel) was included as a positive control for flap endonuclease inhibition.

*FEN1* (Flap ENdonuclease 1) encodes an enzyme previously shown to be amenable to biochemical assay development *in vitro* [25] that has been implicated in almost all DNA transactions, including DNA repair and replication [26]). Adapting a previous radiolabel-based *in vitro* assay, we developed an *in vitro* assay for FEN1 activity based on fluorescence quenching [25]). In this assay, three oligonucleotides are annealed to generate the synthetic substrate, positioning a fluorophore and fluorescent quencher in close proximity. The flap endonuclease activity of FEN1 cleaves the 5′ flap to which the fluorophore is attached, allowing it to diffuse away from the quencher and fluoresce (Figure 2). Using a potent, previously described *in vitro* FEN1 inhibitor, compound 16 from Tumey, *et al.* [27], we observed significant inhibition of flap endonuclease activity (Figure 3, upper left panel). A screen of 30 000 compounds, from libraries containing known and FDA-approved drugs, and the Canadian Chemical Biology Network library, yielded approximately 90 hits, following a counterscreen using a quencherless substrate to eliminate false positives caused by fluorescent compounds and fluorescent quenchers. Ultimately, 13 compounds were selected for further investigation based on structural diversity and having drug-like properties (as described by Lipinski's “Rule of Five”; [28]). These compounds were found to have mid-nanomolar to low micromolar IC_50_s *in vitro* (Figure 3, remaining panels).

### Flap endonuclease inhibitors recapitulate genetic interactions in cells

**Figure 4:**
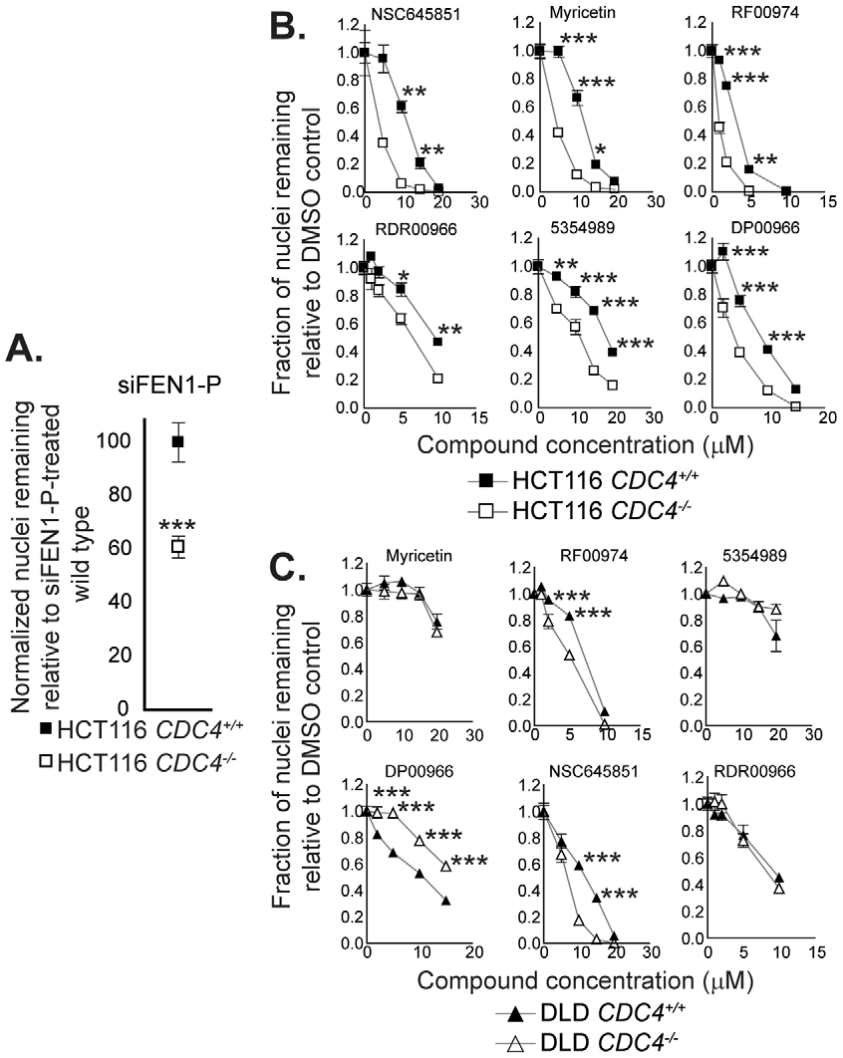
Cell-based assays for flap endonuclease inhibitor activity reveal two compounds that selectively inhibit the proliferation of cells deficient in *CDC4*. (A) siRNA-mediated knockdown of FEN1 selectively inhibits the proliferation of *CDC4*-knockout HCT116 cells. siRNA transfections were carried out as described in Materials and Methods. Cells were fixed and imaged four days following siRNA transfection. Data were analyzed by one-way ANOVA followed by a Tukey test. Shown is mean ± SEM. * p<0.05; ** p<0.01; *** p<0.001. (B) Some flap endonuclease inhibitors recapitulate the genetic interaction between *FEN1* and *CDC4* in HCT116 cells. Cells were incubated with compound at the indicated concentration for 72 hours in optically clear 96-well plates prior to fixation and imaging as described in Materials and Methods. Data were analyzed by one-way ANOVA followed by a Tukey test. Shown is mean ± SEM. * p<0.05; ** p<0.01; *** p<0.001. (C) RF00974 and NSC645851 recapitulate the genetic interaction between *FEN1* and *CDC4* in DLD-1 cells. Experiments were carried out as in (B). Data were analyzed by one-way ANOVA followed by a Tukey test. Shown is mean ± SEM. * p<0.05; ** p<0.01; *** p<0.001.

We next sought to determine whether the flap endonuclease inhibitors we identified could recapitulate any of the genetic interactions found previously (Figure 1F). We first targeted the interaction between *FEN1* and *CDC4*, owing to the fact that *CDC4* has been shown to be a CIN gene mutated in many tumor types [11], [29]–[32]. We took advantage of a matched pair of cell lines in which both copies of CDC4 had been inactivated in HCT116 cells [13]. siRNA-mediated knockdown of FEN1 in this cell pair resulted in selective proliferation inhibition (Figure 4A). We applied the small-molecule hits from the screen to this matched pair of cell lines and found six compounds that selectively inhibited the proliferation of *CDC4*-knockout HCT116 cells relative to wild type cells (Figure 4B and Figure S2B). To ensure that these results were not cell line-specific, we utilized another matched pair of cell lines with inactivated *CDC4*, this time in DLD-1 cells. The six compounds showing selective proliferation inhibition of *CDC4*-knockout HCT116 cells were applied to *CDC4*-knockout and wild type DLD-1 cells [13], and RF00974 and NSC645851 were found to selectively inhibit the proliferation of *CDC4*-knockout DLD-1 cells relative to wild type (Figure 4C).

**Figure 5:**
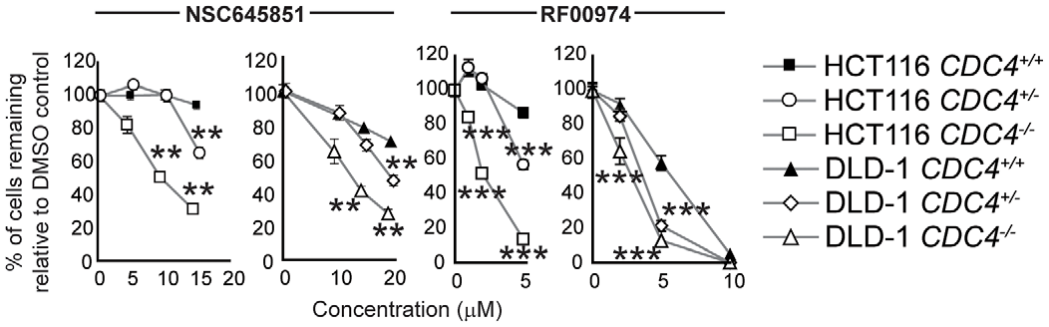
NSC645851 and RF00974 selectively inhibit the proliferation of HCT116 and DLD-1 cells with both homozygous and heterozygous inactivating mutations of *CDC4*. Experiments were carried out as described in Figure 4B. Data were analyzed by one-way ANOVA followed by a Tukey test. Shown is mean ± SEM. * p<0.05; ** p<0.01; *** p<0.001.

To further test the idea that *CDC4* activity is responsible for the observed effect, cells in which *CDC4* had been inactivated in a heterozygous state were also treated with RF00974 and NSC645851. As with homozygous *CDC4^−/−^* cells, heterozygous *CDC4^+/−^* cells displayed a statistically significant decrease in proliferation relative to wild type *CDC4^+/+^* cells, albeit lesser in magnitude (Figure 5).

**Figure 6:**
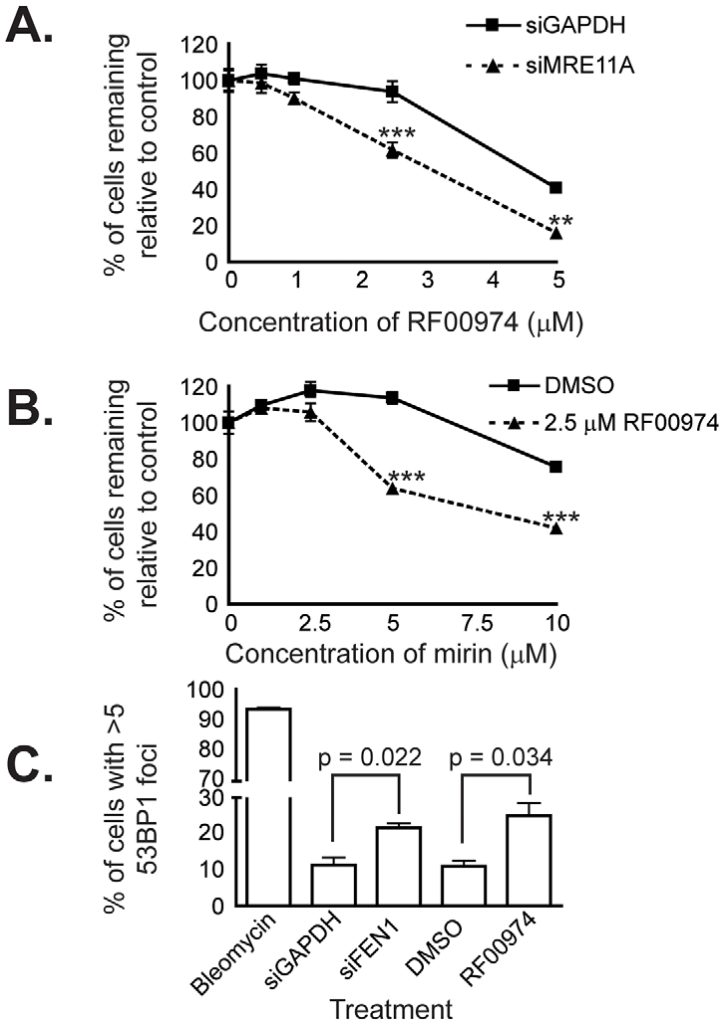
RF00974 recapitulates the interaction between *FEN1* and *MRE11A*, and leads to increased endogenous DNA damage. (A) siRNA-mediated knockdown of MRE11A sensitizes HCT116 cells to treatment with RF00974. siRNA transfection experiments were carried out as described in Materials and Methods. Cells were fixed and imaged four days following siRNA transfection, after cells had been incubated in compound for 48 hours. Data were analyzed by one-way ANOVA followed by a Tukey test. Shown is mean ± SEM. * p<0.05; ** p<0.01; *** p<0.001. (B) Chemical inhibition of MRE11A sensitizes cells to RF00974. Cells were incubated in the indicated compounds at the indicated concentrations for three days prior to fixation and imaging as described in Materials and Methods. Data were analyzed by one-way ANOVA followed by a Tukey test. Shown is mean ± SEM. * p<0.05; ** p<0.01; *** p<0.001. (C) Inhibition of flap endonuclease activity with RF00974 mimics siRNA-mediated knockdown of FEN1 by increasing endogenous DNA damage. Data were analyzed by Student's *t* test. Shown is mean ± SEM.

We next attempted to recapitulate the interaction between *FEN1* and *MRE11A*, as *MRE11A* has been shown to be mutated at a frequency of 4% in colorectal cancers [15]. We treated cells in which MRE11A had been depleted via siRNA with the more potent of the two flap endonuclease inhibitors described above, RF00974, and found that MRE11A depletion sensitized cells to flap endonuclease inhibitor treatment (Figure 6A). We also found that treatment with a previously-described small-molecule inhibitor of MRE11A, mirin [33], was able to sensitize cells to treatment with RF00974 (Figure 6B). Taken together, these data suggest that inhibition of flap endonuclease activity is sufficient to recapitulate evolutionarily conserved, colorectal cancer-relevant synthetic lethal genetic interactions.

Finally, we wished to characterize the mechanism by which inhibition of flap endonuclease activity may lead to cell death. Given the role of FEN1 in DNA replication and repair, we asked whether endogenous DNA damage increases as a result of FEN1 inhibition. We used HCT116 cells in which 53BP1 had been stably tagged with mCherry to ask whether 53BP1 focus formation, indicative of DNA repair centers [34], [35], increased. We found a statistically significant (p<0.05) increase in the frequency of cells with many 53BP1 foci following siRNA-mediated knockdown of FEN1. Furthermore, we observed a similar increase (p<0.05) following treatment with the flap endonuclease inhibitor RF00974 (Figure 6C). We next measured the level of H2AX phosphorylation (γ-H2AX), an independent indicator of DNA damage [36], in HCT116 *CDC4^+/+^* and *CDC4^−/−^* cells in response to RF00974. We found that, similar to increasing 53BP1 focus formation, RF00974 treatment increased H2AX phosphorylation (Figure S3). H2AX phosphorylation was increased even in untreated HCT116 *CDC4^−/−^* cells, so no increase in phosphorylation was observed. In order to determine whether RF00974 leads to an increase in apoptosis in *CDC4*-deficient cells, we asked whether PARP cleavage, a marker of apoptosis [37], is increased following RF00974 treatment. We found that RF00974 treatment did not increase PARP cleavage in either wild type or *CDC4*-deficient cells. Taken together, these results suggest that loss of FEN1, or inhibition of flap endonuclease activity, lead to an increase in endogenous DNA damage that inhibits the proliferation of *CDC4*-deficient cells by non-apoptotic means.

## Discussion

In this study, we used a cross-species candidate approach to identify new anticancer therapeutic targets for small-molecule inhibition having a potentially broad spectrum of applicability. We found that a yeast CIN synthetic lethal interaction network is largely conserved between *S. cerevisiae* and a human tumor cell line. Based on this network, we screened for *in vitro* inhibitors of the highly connected enzyme FEN1. Flap endonuclease inhibitors discovered in this screen recapitulated synthetic lethal interactions between *FEN1* and each of *CDC4* and *MRE11A*, demonstrating that evolutionarily conserved genetic interactions in a core cellular process, such as the maintenance of genomic stability, can be exploited as a means to inhibit the proliferation of tumor cells carrying specific and cancer-relevant mutations.

The idea of using the unique genetic profile of tumor cells relative to somatic cells to selectively kill cancer has been applied by various groups, such as in the case of the chemical-genetic interaction between *BRCA1/2* and PARP inhibitors [2], [3]. Several studies have focused on DNA damage, usually by identifying inhibitors of DNA damage response proteins that either directly kill tumor cells, or that potentiate the effects of DNA damaging agents [38]–[42]. Recently, two large-scale studies examining chemical-genetic interactions between new or established anti-cancer treatments and cancer cell lines of known genotype demonstrated the promise of such top-down approaches by identifying previously unknown sensitivities of many cancer genotypes, such as between Ewing's sarcomas and PARP inhibitors [5], [6]. An alternative means to construct genetic interaction networks for the discovery of therapeutic targets is to take a cross-species candidate approach in a genetically tractable model organism. In *S. cerevisiae*, defined genetic changes can be introduced and subsequently screened in a high-throughput manner [16], [43] (though mammalian genome editing technologies are advancing rapidly [44], [45]). The nearly 75% (16/22) conservation of synthetic lethal interactions we found between yeast and human cells is similar to the degree of conservation of genetic interactions between *S. cerevisiae* and the model metazoan *Caenorhabditis elegans* in a related network, identified by our group and others [18], [19], [46], and expands upon previous proof-of-principle work by our group [47]. Although we ultimately targeted the highly conserved flap endonuclease FEN1 in the current study, yeast genetic data has the potential to implicate biological processes, as opposed to specific proteins, as therapeutic targets; in this way, targets can be identified that are not conserved in *S. cerevisiae*. For example, we recently demonstrated that mutation of cohesin genes in yeast was synthetic lethal with mutation of proteins playing a role in replication fork stability. siRNA-mediated knockdown of cohesin genes was found to sensitize human cells to inhibition of PARP, a protein involved in replication fork progression, but without a known ortholog in yeast [19]. Thus, the versatility of yeast synthetic lethal networks to predict therapeutic targets makes our approach complementary to large-scale screening for gene-drug interactions [4]–[6].

Therapeutics that target a specific genotype, such as EGFR family inhibitors in the case of *ERBB2* (also known as *HER2*) amplification, produce more significant gene-drug interactions than more general cytotoxic agents [6]; however, the indications for such agents are limited to a handful of genotypes. FEN1 plays a critical role in nearly all DNA transactions, including DNA replication via Okazaki fragment maturation [48], [49], long-patch base excision repair [50], [51], the prevention of trinucleotide repeat expansions [25], [52], and restart of stalled replication forks [53]. Yeast RAD27 is one of the most highly genetically connected genes in the yeast genome (Tables S5 and S6); many of these interactors are CIN genes [11], and many of the corresponding human orthologs may prove to be mutated and cause CIN in tumours. Given that the majority of the genetic interactions were conserved in the CIN synthetic lethal interaction network interrogated here, FEN1 may be a widely applicable target in cancers harboring mutations in a variety of CIN genes. More generally, DNA repair and replication protein inhibitors are being actively developed as anticancer therapeutics [2], [3], [41], [54] and the process of DNA replication forms a genetic hub in *S. cerevisiae* [16], [23], [43], [55]. The critical role of FEN1 in DNA transactions is analogous to that of PARP, a protein playing a role in DNA repair and the protection of stalled DNA replication forks [56], [57]. PARP is synthetic lethal with mutations in *BRCA1/2* [2], [3], and its therapeutic range has been extended more recently to include cells with mutations in *PTEN* [38] and cohesins [19]. Thus, like PARP, FEN1 potentially represents a potent, broadly-applicable target for anticancer therapeutic development.

In turn, the ideal anticancer therapeutic would have a broad spectrum, suggesting it would be more advantageous to target a phenotype common in cancer. CIN in the form of aneuploidy is seen in >90% of solid tumors [10] and represents a sub-lethal mutation in an otherwise essential process. Of relevance to the current work, moderate aneuploidy and CIN correlate with poor prognosis in cancer, but extreme aneuploidy correlates with improved patient outcomes [58], [59]. Yeast *RAD27* is a CIN gene [17], and FEN1 mutation in various systems leads to CIN and has been associated with cancer [17], [60]; thus, inhibition of FEN1 in cancers that already exhibit CIN could lead to a level of CIN incompatible with viability. In the present study, flap endonuclease inhibitors were found to recapitulate the synthetic lethal interactions between *FEN1* and each of *CDC4* and *MRE11A* [18], [23]. We observed that both depletion and inhibition of flap endonuclease activity led to an increase in endogenous DNA damage. Recent reports have shown that γ-H2AX levels are not increased following FEN1 depletion [61]; however, we observed increases in DNA damage using two independent assays following two means of FEN1 inhibition, and attribute these results to cell background differences, such as the mismatch repair deficiency present in HCT116 cells. Furthermore, this increase in DNA damage led to a non-apoptotic inhibition of proliferation. Thus, one explanation for the lethality in combination with inactivation of *CDC4* is that the cell is inappropriately driven through the cell cycle, owing to elevated levels of cyclin E [13], when otherwise it would arrest to try to repair DNA damage. Likewise, increased endogenous DNA damage combined with loss of MRE11A, a protein playing a critical role in the first steps of the DNA damage response [62], could lead to a level of DNA damage or mutation that is incompatible with proliferation. *CDC4* has been reported to be mutated in a wide variety of tumor types, at frequencies ranging from 6% to >30%, depending on the tumor type [13], [21], [29], [63], [64]. Recently, it has been suggested that reduction of *CDC4* activity to some level below that of wild type, but above complete abrogation of function, is optimal for tumor progression [63]. Thus, the fact that two flap endonuclease inhibitors described here were able to selectively inhibit the proliferation of both heterozygous and homozygous *CDC4*-knockout cell lines suggests that CDC4 loss, whether complete or partial, sensitizes cells to inhibition of flap endonuclease activity. As well, the fact that both genotypes were sensitive to inhibition of flap endonuclease activity adds weight to the suggestion that this response is specific to CDC4 activity, in the same way that changing response following alteration in dosage in biochemical screening is suggestive of target identity [65].

In summary, here we have presented a rational, cross-species approach to the identification of anticancer therapeutic targets by targeting CIN, a common cancer phenotype. The use of conserved synthetic lethal interaction networks to identify highly-connected second-site targets is an accessible alternative to large scale screens: it narrows down the number of synthetic lethal gene pairs to be directly retested from tens of thousands to dozens, and is based on strong synthetic lethal interactions discovered in yeast networks. We have demonstrated the potential of this approach to identify targets and therapeutics, such as FEN1 and the flap endonuclease inhibitors described here, having potentially broad applicability in the treatment of cancer.

## Materials and Methods

### Cell culture

HCT116 cells were purchased from ATCC. HCT116 derivatives, DLD-1 and DLD-1 derivatives were gifts of Dr. Bert Vogelstein (Johns Hopkins University). (Importantly, we observed that the deleted exon in *CDC4* in these cell lines is not exon 5, as previously reported [13], but exon 8. We attribute the difference to changing annotations in public sequence databases between 2004 and the present.) 53BP1-mCherry HCT116 cells were a gift of Dr. Sam Aparicio (UBC). These cells were grown in McCoy's 5A medium with 10% FBS. Immortalized (telomerase) BJ normal human skin fibroblasts, hTERT [66], were generously provided by Dr. C.P. Case (University of Bristol) and were grown in DMEM containing 10% FBS. Mirin was purchased from Sigma-Aldrich. RF00974 was purchased from Maybridge, Ltd.

### Western blotting

Western blots were performed as detailed elsewhere [47]. Antibodies used for Western blots are described in Table S4.

### RNA interference

Subconfluent and asynchronous cells were transiently transfected with siRNAs. HCT116 cells were transfected with ON-TARGET*plus* siRNA pools at a total siRNA concentration of 25 nM using DharmaFECT I (Dharmacon). In dual siRNA experiments, the total siRNA concentration was 50 nM. Cultures were replenished with fresh medium 11 hours after transfection. hTERT cells were transfected with ON-TARGET*plus* siRNA pools, or independent duplexes, at a total siRNA concentration of 100 nM using RNAiMax (Invitrogen). Cultures were replenished with fresh medium 24 hours after transfection.

### Synthetic lethal assays, cell imaging, and compound incubation

HCT116 cells were harvested 24 hours after siRNA transfection and re-plated in 96-well optical bottom plates. hTERT cells were transfected directly in 96-well plates. HCT116 cells were fixed four days after transfection, and hTERT cells were fixed seven days after transfection, in 4% paraformaldehyde/PBS. Nuclei were labelled with Hoechst 33342. Stained nuclei were counted using a Cellomics Arrayscan VTI fluorescence imager as described previously [47] or a Zeiss AxioObserver Z1 equipped with an LED Colibri light source, a 20× plan apochromat dry lens (numerical aperture = 0.8) and AxioVision v4.8 software. Images were analyzed using the Physiology Analyzer (Assaybuilder) option within the AxioVision software. Data were normalized to GAPDH-silenced controls and conventional statistics (e.g. column statistics and Student's *t*-tests) were performed. Experiments were performed twice; indicated numbers are averaged from at least 6 wells.

To determine the presence of a synthetic lethal interaction, the proliferative defect was calculated, and is defined as
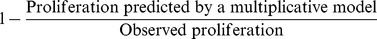
where the predicted proliferation was the product of the proliferation of the two individual gene knockdowns, following a multiplicative model of genetic interactions [67]. Synthetic lethal interactions were scored as a proliferative defect of three times the average SEM of the experiment or greater.

During compound incubation experiments, cells were incubated in compound of interest in 96-well optical bottom plates for approximately three days prior to fixation and analysis. Data (from six independent wells) were analyzed using a one-way ANOVA followed by a Tukey test.

### FEN1 purification

FEN1 was expressed in BL21 *E. coli* from pET28b(+) (a generous gift from R. Bambara, University of Rochester) using 1 mM IPTG. Bacteria were lysed in lysis buffer (50 mM NaH_2_PO_4_, 300 mM NaCl, 10 mM imidazole, pH 8.0 containing 2× protease inhibitor) via a French press at 10 000 psi. The lysate was clarified and passed through a 0.22 µM filter before being loaded onto a HisTrap FF column (1 mL, GE Healthcare) in an ÄKTAFPLC P-920 system (GE Healthcare). The column was washed in 10 volumes of wash buffer (lysis buffer+20 mM imidazole), and FEN1 was eluted with 5 volumes of elution buffer (lysis buffer+125 mM imidazole). The lysate was diluted with 9 volumes HI buffer (30 mM HEPES-KOH, 0.5% *myo*-inositol, pH 7.8) with 30 mM NaH_2_PO_4_ and concentrated in a protein concentrator (Amicon). It was then loaded onto a hydroxyapatite resin (HA Ultrogel, Pall Life Sciences). The hydroxyapatite resin was washed with 10 volumes of HI-30 mM PO_4_, and FEN1 was eluted with 5 volumes of HI-200 mM PO_4_. The eluate was diluted with 5 volumes HI-30 mM KCl prior to concentration, and then loaded onto a strong cation exchange column (1 mL HiTRAP SP FF FPLC, GE Healthcare Life Sciences). The column was washed with 10 volumes of HI-30 mM KCl, then 10 volumes of HI-200 mM KCl, and FEN1 was eluted with a gradient from HI-200 mM KCl to HI-500 mM KCl over 10 column volumes. Purified FEN1 was concentrated in FEN1 dilution buffer (30 mM HEPES-KOH, 5% glycerol, 0.1 mg/mL BSA, 0.01% NP-40), and aliquots of known concentration were frozen at −80°C.

### *In vitro* FEN1 inhibition assay

Oligonucleotides used were as follows: “template”, 5′-GGTGGACGGGTGGATTGAAATTTAGGCTGGCACGGTCG-3′, “upstream”, 5′-CGACCGTGCCAGCCTAAATTTCAATC-3′, “downstream”, 5′-*6-FAM*-CCAAGGCCACCCGTCCAC-*BHQ-1*-3′. (6-FAM is 6-carboxyfluorescein; BHQ-1 is black hole quencher 1.) The three oligonucleotides were annealed at equimolar amounts in annealing buffer (50 mM Tris, 50 mM NaCl, 1 mM DTT, pH 8.0) by heating to 94°C, cooling to 70°C, and gradually cooling to room temperature. FEN1 assays were carried out with 6 pmol FEN1 and 20 nM annealed substrate in FEN1 buffer (50 mM Tris pH 8.0, 30 mM NaCl, 8 mM MgCl_2_, 0.1 mg/mL BSA, 2 mM DTT). Assays were carried out at room temperature and kinetic reads were taken over approximately ten minutes in a Varioskan plate reader (Thermo Fisher Scientific), using excitation and emission wavelengths of 492 nm and 517 nm, respectively.

### Fluorescent imaging

53BP1-mCherry cells were grown on cover slips. Following desired treatment (either two hours of bleomycin treatment at 5 µg/mL, four days following siRNA transfection, or after 24 hours of RF00974 treatment at 10 µM), cells were fixed for five minutes in 4% paraformaldehyde/PBS, mounted in Vectashield mounting medium containing DAPI (500 ng/mL), and imaged on a Zeiss Axioplan microscope with a Coolsnap HQ camera, using appropriate filters and controlled by Metamorph software.

### Apoptosis analysis

Cells were treated with RF00974 for 48 hours prior to harvesting of medium and cells in lysis buffer (50 mM Tris, 150 mM NaCl, 1% Triton-X-100, pH 7.5). Lysates were sonicated and clarified by centrifugation at 13 000 rpm for 15 minutes at 4°C. As a positive control, HCT116 cells were treated with 1 µM staurosporine prior to harvesting. Lysates were subjected to Western blotting as described above.

### Synthetic genetic array

Synthetic genetic array analysis of *rad27Δ* against a collection of yeast essential DAmP alleles [68] and temperature sensitive alleles [69] was carried out as described previously [11], [19].

## Supporting Information

Figure S1 Western blots demonstrating knockdown of gene products targeted in this study. Cells were transfected with siRNA SMARTpools targeting the genes of interest. Proteins were harvested 3 days after transfection and Western blots were performed as detailed in Materials and Methods. Anti-α-tubulin was used as a loading control. (A) Knockdown of siRNA pools in HCT116 cells. (B) Knockdown of individual siRNA duplexes in hTERT cells.
(TIF)

Figure S2 (A) The effect of non-silencing siRNA versus GAPDH siRNA on HCT116 cells. Cells were transfected with the indicated siRNAs, transferred to 96-well plates, fixed, and imaged as in Materials and Methods. (B) Response of HCT116 cells to selected compounds. Cells were incubated with compound at the indicated concentration for 72 hours in optically clear 96-well plates prior to fixation and imaging as described in Materials and Methods. Data were analyzed by one-way ANOVA followed by a Tukey test. Shown is mean ± SEM.
(TIF)

Figure S3 Treatment of cells with RF00974 leads to an increase in phosphorylated H2AX, but not to an increase in apoptosis. Cells were treated with the indicated concentrations of RF00974 for 48 hours before protein was harvested and subjected to Western blot. Staurosporine (Stau) was used as a control to initiate apoptosis.
(TIF)

Table S1 siRNA pool silencing in HCT116 cells. Horizontal lines indicate experiments carried out on different days.
(DOC)

Table S2 siRNA pool silencing in hTERT cells.
(DOC)

Table S3 Synthetic Lethality between FEN1 and cancer genes in hTERT cells. Horizontal lines indicate experiments carried out on different days.
(DOC)

Table S4 Antibodies employed in Western blots in this study.
(DOC)

Table S5 Genetic interactors of *rad27Δ* and cancer mutations.
(XLS)

Table S6 Raw data from *rad27Δ* SGA against a collection temperature-sensitive and DAmP alleles of essential genes. #Spots, number of times allele was represented on array. E-C, experimental value minus control value (negative values indicate double mutant grows more slowly than control). Pval, p value of E-C.
(XLS)

## References

[1] Hartwell LH, Szankasi P, Roberts CJ, Murray AW, Friend SH (1997) Integrating genetic approaches into the discovery of anticancer drugs. Science 278: 1064–1068 10.1126/science.278.5340.1064. 9353181 10.1126/science.278.5340.1064

[2] Farmer H, McCabe N, Lord CJ, Tutt AN, Johnson DA, et al (2005) Targeting the DNA repair defect in BRCA mutant cells as a therapeutic strategy. Nature 434: 917–921. 15829967 10.1038/nature03445

[3] Bryant HE, Schultz N, Thomas HD, Parker KM, Flower D, et al (2005) Specific killing of BRCA2-deficient tumours with inhibitors of poly(ADP-ribose) polymerase. Nature 434: 913–917. 15829966 10.1038/nature03443

[4] Weinstein JN, Myers TG, O'Connor PM, Friend SH, Fornace AJ Jr, et al (1997) An information-intensive approach to the molecular pharmacology of cancer. Science 275: 343–349. 8994024 10.1126/science.275.5298.343

[5] Barretina J, Caponigro G, Stransky N, Venkatesan K, Margolin AA, et al (2012) The Cancer Cell Line Encyclopedia enables predictive modelling of anticancer drug sensitivity. Nature 483: 603–607. 22460905 10.1038/nature11003 PMC3320027

[6] Garnett MJ, Edelman EJ, Heidorn SJ, Greenman CD, Dastur A, et al (2012) Systematic identification of genomic markers of drug sensitivity in cancer cells. Nature 483: 570–575. 22460902 10.1038/nature11005 PMC3349233

[7] Grebien F, Hantschel O, Wojcik J, Kaupe I, Kovacic B, et al (2011) Targeting the SH2-kinase interface in Bcr-Abl inhibits leukemogenesis. Cell 147: 306–319. 22000011 10.1016/j.cell.2011.08.046 PMC3202669

[8] Zhao C, Chen A, Jamieson CH, Fereshteh M, Abrahamsson A, et al (2009) Hedgehog signalling is essential for maintenance of cancer stem cells in myeloid leukaemia. Nature 458: 776–779. 19169242 10.1038/nature07737 PMC2946231

[9] Hanahan D, Weinberg RA (2011) Hallmarks of cancer: the next generation. Cell 144: 646–674. 21376230 10.1016/j.cell.2011.02.013

[10] Weaver BA, Cleveland DW (2006) Does aneuploidy cause cancer? Curr Opin Cell Biol 18: 658–667. 17046232 10.1016/j.ceb.2006.10.002

[11] Stirling PC, Bloom MS, Solanki-Patil T, Smith S, Sipahimalani P, et al (2011) The complete spectrum of yeast chromosome instability genes identifies candidate CIN cancer genes and functional roles for ASTRA complex components. PLoS Genet 7: e1002057 doi:10.1371/journal.pgen.1002057. 21552543 10.1371/journal.pgen.1002057 PMC3084213

[12] Cahill DP, Lengauer C, Yu J, Riggins GJ, Willson JKV, et al (1998) Mutations of mitotic checkpoint genes in human cancers. Nature 392: 300–303 10.1038/32688. 9521327 10.1038/32688

[13] Rajagopalan H, Jallepalli PV, Rago C, Velculescu VE, Kinzler KW, et al (2004) Inactivation of hCDC4 can cause chromosomal instability. Nature 428: 77–81 10.1038/nature02313. 14999283 10.1038/nature02313

[14] Barber TD, McManus K, Yuen KW, Reis M, Parmigiani G, et al (2008) Chromatid cohesion defects may underlie chromosome instability in human colorectal cancers. Proc Natl Acad Sci U S A 105: 3443–3448. 18299561 10.1073/pnas.0712384105 PMC2265152

[15] Wang Z, Cummins JM, Shen D, Cahill DP, Jallepalli PV, et al (2004) Three classes of genes mutated in colorectal cancers with chromosomal instability. Cancer Res 64: 2998–3001. 15126332 10.1158/0008-5472.can-04-0587

[16] Tong AH, Evangelista M, Parsons AB, Xu H, Bader GD, et al (2001) Systematic genetic analysis with ordered arrays of yeast deletion mutants. Science 294: 2364–2368. 11743205 10.1126/science.1065810

[17] Yuen KW, Warren CD, Chen O, Kwok T, Hieter P, et al (2007) Systematic genome instability screens in yeast and their potential relevance to cancer. Proc Natl Acad Sci U S A 104: 3925–3930. 17360454 10.1073/pnas.0610642104 PMC1820685

[18] McLellan J, O'Neil N, Tarailo S, Stoepel J, Bryan J, et al (2009) Synthetic lethal genetic interactions that decrease somatic cell proliferation in Caenorhabditis elegans identify the alternative RFC CTF18 as a candidate cancer drug target. Mol Biol Cell 20: 5306–5313. 19846659 10.1091/mbc.E09-08-0699 PMC2793303

[19] McLellan JL, O'Neil NJ, Barrett I, Ferree E, van Pel DM, et al (2012) Synthetic lethality of cohesins with PARPs and replication fork mediators. PLoS Genet 8: e1002574 doi:10.1371/journal.pgen.1002574. 22412391 10.1371/journal.pgen.1002574 PMC3297586

[20] Hiramoto T, Nakanishi T, Sumiyoshi T, Fukuda T, Matsuura S, et al (1999) Mutations of a novel human RAD54 homologue, RAD54B, in primary cancer. Oncogene 18: 3422–3426. 10362364 10.1038/sj.onc.1202691

[21] Kemp Z, Rowan A, Chambers W, Wortham N, Halford S, et al (2005) CDC4 mutations occur in a subset of colorectal cancers but are not predicted to cause loss of function and are not associated with chromosomal instability. Cancer Res 65: 11361–11366. 16357143 10.1158/0008-5472.CAN-05-2565

[22] Cahill DP, Kinzler KW, Vogelstein B, Lengauer C (1999) Genetic instability and darwinian selection in tumours. Trends Cell Biol 9: M57–M60 10.1016/S0962-8924(99)01661-X. 10611684

[23] Costanzo M, Baryshnikova A, Bellay J, Kim Y, Spear ED, et al (2010) The genetic landscape of a cell. Science 327: 425–431. 20093466 10.1126/science.1180823 PMC5600254

[24] Prieto I, Suja JA, Pezzi N, Kremer L, Martinez A, et al (2001) Mammalian STAG3 is a cohesin specific to sister chromatid arms in meiosis I. Nat Cell Biol 3: 761–766. 11483963 10.1038/35087082

[25] Liu Y, Bambara RA (2003) Analysis of human flap endonuclease 1 mutants reveals a mechanism to prevent triplet repeat expansion. J Biol Chem 278: 13728–13739. 12554738 10.1074/jbc.M212061200

[26] Zheng L, Jia J, Finger LD, Guo Z, Zer C, et al (2011) Functional regulation of FEN1 nuclease and its link to cancer. Nucleic Acids Res 39: 781–794. 20929870 10.1093/nar/gkq884 PMC3035468

[27] Tumey LN, Bom D, Huck B, Gleason E, Wang J, et al (2005) The identification and optimization of a N-hydroxy urea series of flap endonuclease 1 inhibitors. Bioorg Med Chem Lett 15: 277–281. 15603939 10.1016/j.bmcl.2004.10.086

[28] Lipinski CA, Lombardo F, Dominy BW, Feeney PJ (2001) Experimental and computational approaches to estimate solubility and permeability in drug discovery and development settings. Adv Drug Deliv Rev 46: 3–26. 11259830 10.1016/s0169-409x(00)00129-0

[29] Akhoondi S, Sun D, von der LN, Apostolidou S, Klotz K, et al (2007) FBXW7/hCDC4 is a general tumor suppressor in human cancer. Cancer Res 67: 9006–9012. 17909001 10.1158/0008-5472.CAN-07-1320

[30] Koh JL, Ding H, Costanzo M, Baryshnikova A, Toufighi K, et al (2010) DRYGIN: a database of quantitative genetic interaction networks in yeast. Nucleic Acids Res 38: D502–D507. 19880385 10.1093/nar/gkp820 PMC2808960

[31] Miyaki M, Yamaguchi T, Iijima T, Takahashi K, Matsumoto H, et al (2009) Somatic mutations of the CDC4 (FBXW7) gene in hereditary colorectal tumors. Oncology 76: 430–434. 19420964 10.1159/000217811

[32] Milne AN, Leguit R, Corver WE, Morsink FH, Polak M, et al (2010) Loss of CDC4/FBXW7 in gastric carcinoma. Cell Oncol 32: 347–359. 20448329 10.3233/CLO-2010-523 PMC4619292

[33] Dupre A, Boyer-Chatenet L, Sattler RM, Modi AP, Lee JH, et al (2008) A forward chemical genetic screen reveals an inhibitor of the Mre11-Rad50-Nbs1 complex. Nat Chem Biol 4: 119–125. 18176557 10.1038/nchembio.63 PMC3065498

[34] Anderson L, Henderson C, Adachi Y (2001) Phosphorylation and rapid relocalization of 53BP1 to nuclear foci upon DNA damage. Mol Cell Biol 21: 1719–1729. 11238909 10.1128/MCB.21.5.1719-1729.2001 PMC86718

[35] Rappold I, Iwabuchi K, Date T, Chen J (2001) Tumor suppressor p53 binding protein 1 (53BP1) is involved in DNA damage-signaling pathways. J Cell Biol 153: 613–620. 11331310 10.1083/jcb.153.3.613 PMC2190566

[36] Markova E, Schultz N, Belyaev IY (2007) Kinetics and dose-response of residual 53BP1/gamma-H2AX foci: co-localization, relationship with DSB repair and clonogenic survival. Int J Radiat Biol 83: 319–329. 17457757 10.1080/09553000601170469

[37] Lazebnik YA, Kaufmann SH, Desnoyers S, Poirier GG, Earnshaw WC (1994) Cleavage of poly(ADP-ribose) polymerase by a proteinase with properties like ICE. Nature 371: 346–347. 8090205 10.1038/371346a0

[38] Mendes-Pereira AM, Martin SA, Brough R, McCarthy A, Taylor JR, et al (2009) Synthetic lethal targeting of PTEN mutant cells with PARP inhibitors. EMBO Mol Med 1: 315–322. 20049735 10.1002/emmm.200900041 PMC3378149

[39] Reaper PM, Griffiths MR, Long JM, Charrier JD, Maccormick S, et al (2011) Selective killing of ATM- or p53-deficient cancer cells through inhibition of ATR. Nat Chem Biol 10.1038/nchembio.573 21490603

[40] Lengauer C, Kinzler KW, Vogelstein B (1997) Genetic instability in colorectal cancers. Nature 386: 623–627 10.1038/386623a0. 9121588 10.1038/386623a0

[41] Feng Z, Scott SP, Bussen W, Sharma GG, Guo G, et al (2011) Rad52 inactivation is synthetically lethal with BRCA2 deficiency. Proc Natl Acad Sci U S A 108: 686–691. 21148102 10.1073/pnas.1010959107 PMC3021033

[42] Martin SA, McCabe N, Mullarkey M, Cummins R, Burgess DJ, et al (2010) DNA polymerases as potential therapeutic targets for cancers deficient in the DNA mismatch repair proteins MSH2 or MLH1. Cancer Cell 17: 235–248. 20227038 10.1016/j.ccr.2009.12.046 PMC2845806

[43] Pan X, Yuan DS, Xiang D, Wang X, Sookhai-Mahadeo S, et al (2004) A robust toolkit for functional profiling of the yeast genome. Mol Cell 16: 487–496 10.1016/j.molcel.2004.09.035. 15525520 10.1016/j.molcel.2004.09.035

[44] Le PF, Lillico S, Passet B, Young R, Whitelaw B, et al (2010) Zinc finger nuclease technology heralds a new era in mammalian transgenesis. Trends Biotechnol 28: 134–141. 20015561 10.1016/j.tibtech.2009.11.007

[45] Cermak T, Doyle EL, Christian M, Wang L, Zhang Y, et al (2011) Efficient design and assembly of custom TALEN and other TAL effector-based constructs for DNA targeting. Nucleic Acids Res 39: e82. 21493687 10.1093/nar/gkr218 PMC3130291

[46] Tarailo M, Tarailo S, Rose AM (2007) Synthetic lethal interactions identify phenotypic “interologs” of the spindle assembly checkpoint components. Genetics 177: 2525–2530. 18073444 10.1534/genetics.107.080408 PMC2219473

[47] McManus KJ, Barrett IJ, Nouhi Y, Hieter P (2009) Specific synthetic lethal killing of RAD54B-deficient human colorectal cancer cells by FEN1 silencing. Proc Natl Acad Sci U S A 106: 3276–3281. 19218431 10.1073/pnas.0813414106 PMC2651317

[48] Murante RS, Rumbaugh JA, Barnes CJ, Norton JR, Bambara RA (1996) Calf RTH-1 nuclease can remove the initiator RNAs of Okazaki fragments by endonuclease activity. J Biol Chem 271: 25888–25897. 8824221 10.1074/jbc.271.42.25888

[49] Harrington JJ, Lieber MR (1994) The characterization of a mammalian DNA structure-specific endonuclease. EMBO J 13: 1235–1246. 8131753 10.1002/j.1460-2075.1994.tb06373.x PMC394933

[50] Kim K, Biade S, Matsumoto Y (1998) Involvement of flap endonuclease 1 in base excision DNA repair. J Biol Chem 273: 8842–8848. 9535864 10.1074/jbc.273.15.8842

[51] Klungland A, Lindahl T (1997) Second pathway for completion of human DNA base excision-repair: reconstitution with purified proteins and requirement for DNase IV (FEN1). EMBO J 16: 3341–3348. 9214649 10.1093/emboj/16.11.3341 PMC1169950

[52] Singh P, Zheng L, Chavez V, Qiu J, Shen B (2007) Concerted action of exonuclease and Gap-dependent endonuclease activities of FEN-1 contributes to the resolution of triplet repeat sequences (CTG)n- and (GAA)n-derived secondary structures formed during maturation of Okazaki fragments. J Biol Chem 282: 3465–3477. 17138563 10.1074/jbc.M606582200

[53] Zheng L, Zhou M, Chai Q, Parrish J, Xue D, et al (2005) Novel function of the flap endonuclease 1 complex in processing stalled DNA replication forks. EMBO Rep 6: 83–89. 15592449 10.1038/sj.embor.7400313 PMC1299223

[54] Jaiswal AS, Banerjee S, Aneja R, Sarkar FH, Ostrov DA, et al (2011) DNA Polymerase beta as a Novel Target for Chemotherapeutic Intervention of Colorectal Cancer. PLoS ONE 6: e16691 doi:10.1371/journal.pone.0016691. 21311763 10.1371/journal.pone.0016691 PMC3032781

[55] Tong AH, Lesage G, Bader GD, Ding H, Xu H, et al (2004) Global mapping of the yeast genetic interaction network. Science 303: 808–813 10.1126/science.1091317. 14764870 10.1126/science.1091317

[56] Strom CE, Johansson F, Uhlen M, Szigyarto CA, Erixon K, et al (2011) Poly (ADP-ribose) polymerase (PARP) is not involved in base excision repair but PARP inhibition traps a single-strand intermediate. Nucleic Acids Res 39: 3166–3175. 21183466 10.1093/nar/gkq1241 PMC3082910

[57] Ying S, Hamdy FC, Helleday T (2012) Mre11-dependent degradation of stalled DNA replication forks is prevented by BRCA2 and PARP1. Cancer Res 72: 2814–2821. 22447567 10.1158/0008-5472.CAN-11-3417

[58] Birkbak NJ, Eklund AC, Li Q, McClelland SE, Endesfelder D, et al (2011) Paradoxical relationship between chromosomal instability and survival outcome in cancer. Cancer Res 71: 3447–3452. 21270108 10.1158/0008-5472.CAN-10-3667 PMC3096721

[59] Lee AJ, Endesfelder D, Rowan AJ, Walther A, Birkbak NJ, et al (2011) Chromosomal instability confers intrinsic multidrug resistance. Cancer Res 71: 1858–1870. 21363922 10.1158/0008-5472.CAN-10-3604 PMC3059493

[60] Zheng L, Dai H, Hegde ML, Zhou M, Guo Z, et al (2011) Fen1 mutations that specifically disrupt its interaction with PCNA cause aneuploidy-associated cancer. Cell Res 10.1038/cr.2011.35 PMC3129403 21383776

[61] Duxin JP, Moore HR, Sidorova J, Karanja K, Honaker Y, et al (2012) An Okazaki fragment processing-independent role for human Dna2 during DNA replication. J Biol Chem 10.1074/jbc.M112.359018 PMC3381158 22570476

[62] Stracker TH, Petrini JH (2011) The MRE11 complex: starting from the ends. Nat Rev Mol Cell Biol 12: 90–103. 21252998 10.1038/nrm3047 PMC3905242

[63] Davis H, Tomlinson I (2012) CDC4/FBXW7 and the ‘just enough’ model of tumourigenesis. J Pathol 227: 131–135. 22323043 10.1002/path.4004 PMC4594772

[64] Le GM, O'Hara AJ, Rudd ML, Urick ME, Hansen NF, et al (2012) Exome sequencing of serous endometrial tumors identifies recurrent somatic mutations in chromatin-remodeling and ubiquitin ligase complex genes. Nat Genet 10. 10.1038/ng.2455 PMC3515204 23104009

[65] Hoon S, Smith AM, Wallace IM, Suresh S, Miranda M, et al (2008) An integrated platform of genomic assays reveals small-molecule bioactivities. Nat Chem Biol 4: 498–506. 18622389 10.1038/nchembio.100

[66] Cogan N, Baird DM, Phillips R, Crompton LA, Caldwell MA, et al (2010) DNA damaging bystander signalling from stem cells, cancer cells and fibroblasts after Cr(VI) exposure and its dependence on telomerase. Mutat Res 683: 1–8. 19800897 10.1016/j.mrfmmm.2009.09.012

[67] Baryshnikova A, Costanzo M, Dixon S, Vizeacoumar FJ, Myers CL, et al (2010) Synthetic genetic array (SGA) analysis in Saccharomyces cerevisiae and Schizosaccharomyces pombe. Methods Enzymol 470: 145–79 Epub;%2010 Mar 1.: 145–179. 20946810 10.1016/S0076-6879(10)70007-0

[68] Breslow DK, Cameron DM, Collins SR, Schuldiner M, Stewart-Ornstein J, et al (2008) A comprehensive strategy enabling high-resolution functional analysis of the yeast genome. Nat Methods 5: 711–718. 18622397 10.1038/nmeth.1234 PMC2756093

[69] Li Z, Vizeacoumar FJ, Bahr S, Li J, Warringer J, et al (2011) Systematic exploration of essential yeast gene function with temperature-sensitive mutants. Nat Biotechnol 29: 361–367. 21441928 10.1038/nbt.1832 PMC3286520

