# Correction: An Evolutionarily Conserved Synthetic Lethal Interaction Network Identifies FEN1 as a Broad-Spectrum Target for Anticancer Therapeutic Development

**DOI:** 10.1371/annotation/a26cb527-ec18-46ec-a63f-c69d0983add6

**Published:** 2013-02-18

**Authors:** Derek M. van Pel, Irene J. Barrett, Yoko Shimizu, Babu V. Sajesh, Brent J. Guppy, Tom Pfeifer, Kirk J. McManus, Philip Hieter

The affiliation for the fourth and fifth authors was incorrect. Babu V. Sajesh and Brent J. Guppy are not affiliated with the Department of Screening, Centre for Drug Research and Development, Vancouver, Canada, but are affiliated with the Manitoba Institute of Cell Biology, Department of Biochemistry and Medical Genetics, University of Manitoba, Winnipeg, Canada.

