# Supplementary figures and images for: An Evolutionarily Conserved Synthetic Lethal Interaction Network Identifies FEN1 as a Broad-Spectrum Target for Anticancer Therapeutic Development
Source: PLoS Genet. 2013 Jan 31;9(1):e1003254. doi: 10.1371/journal.pgen.1003254 (PMC3561056; doi:10.1371/journal.pgen.1003254)

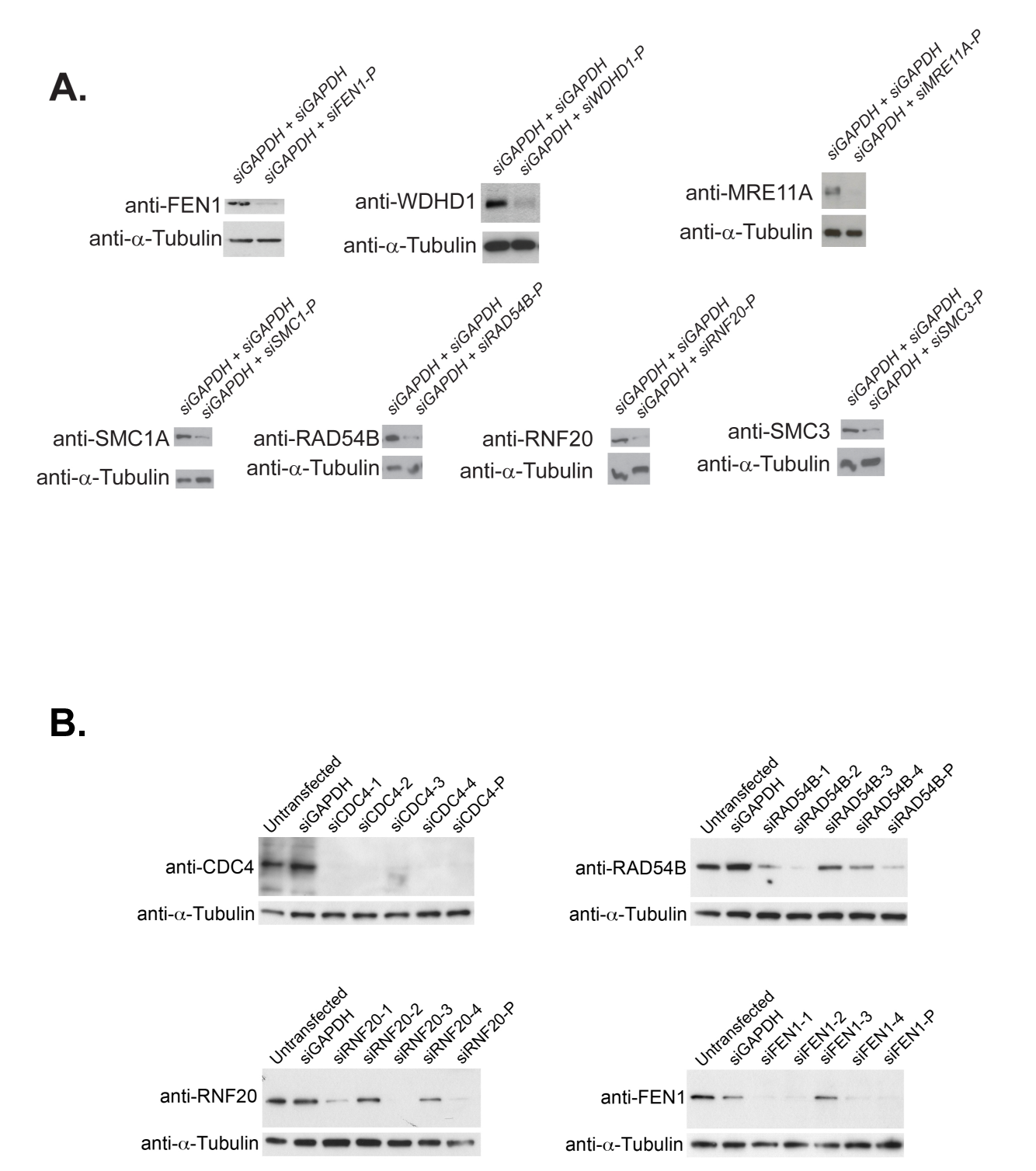

Supplement: Figure S1 — Western blots demonstrating knockdown of gene products targeted in this study. Cells were transfected with siRNA SMARTpools targeting the genes of interest. Proteins were harvested 3 days after transfection and Western blots were performed as detailed in Materials and Methods. Anti-α-tubulin was used as a loading control. (A) Knockdown of siRNA pools in HCT116 cells. (B) Knockdown of individual siRNA duplexes in hTERT cells. (TIF) [file pgen.1003254.s001.tif]

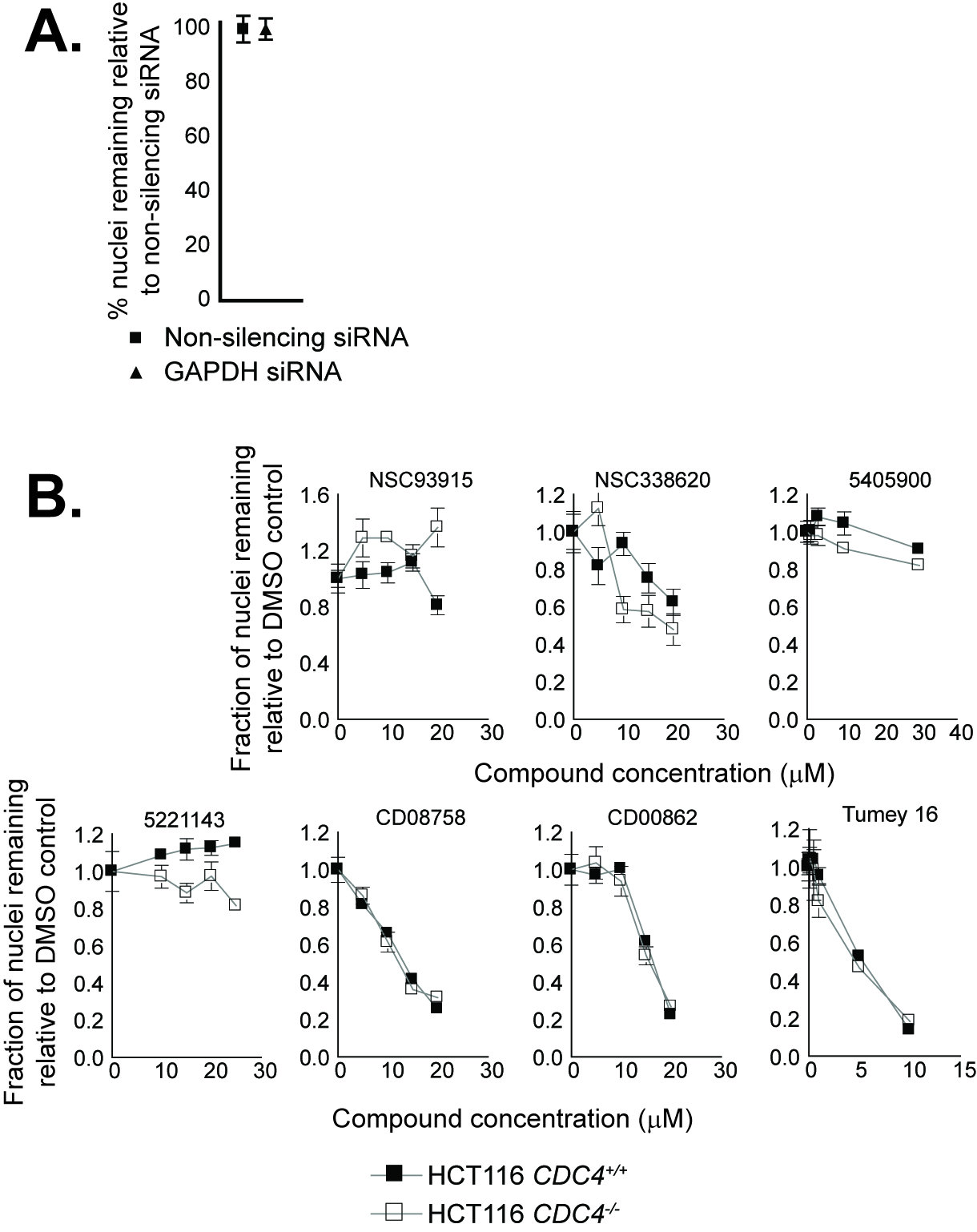

Supplement: Figure S2 — (A) The effect of non-silencing siRNA versus GAPDH siRNA on HCT116 cells. Cells were transfected with the indicated siRNAs, transferred to 96-well plates, fixed, and imaged as in Materials and Methods. (B) Response of HCT116 cells to selected compounds. Cells were incubated with compound at the indicated concentration for 72 hours in optically clear 96-well plates prior to fixation and imaging as described in Materials and Methods. Data were analyzed by one-way ANOVA followed by a Tukey test. Shown is mean ± SEM. (TIF) [file pgen.1003254.s002.tif]

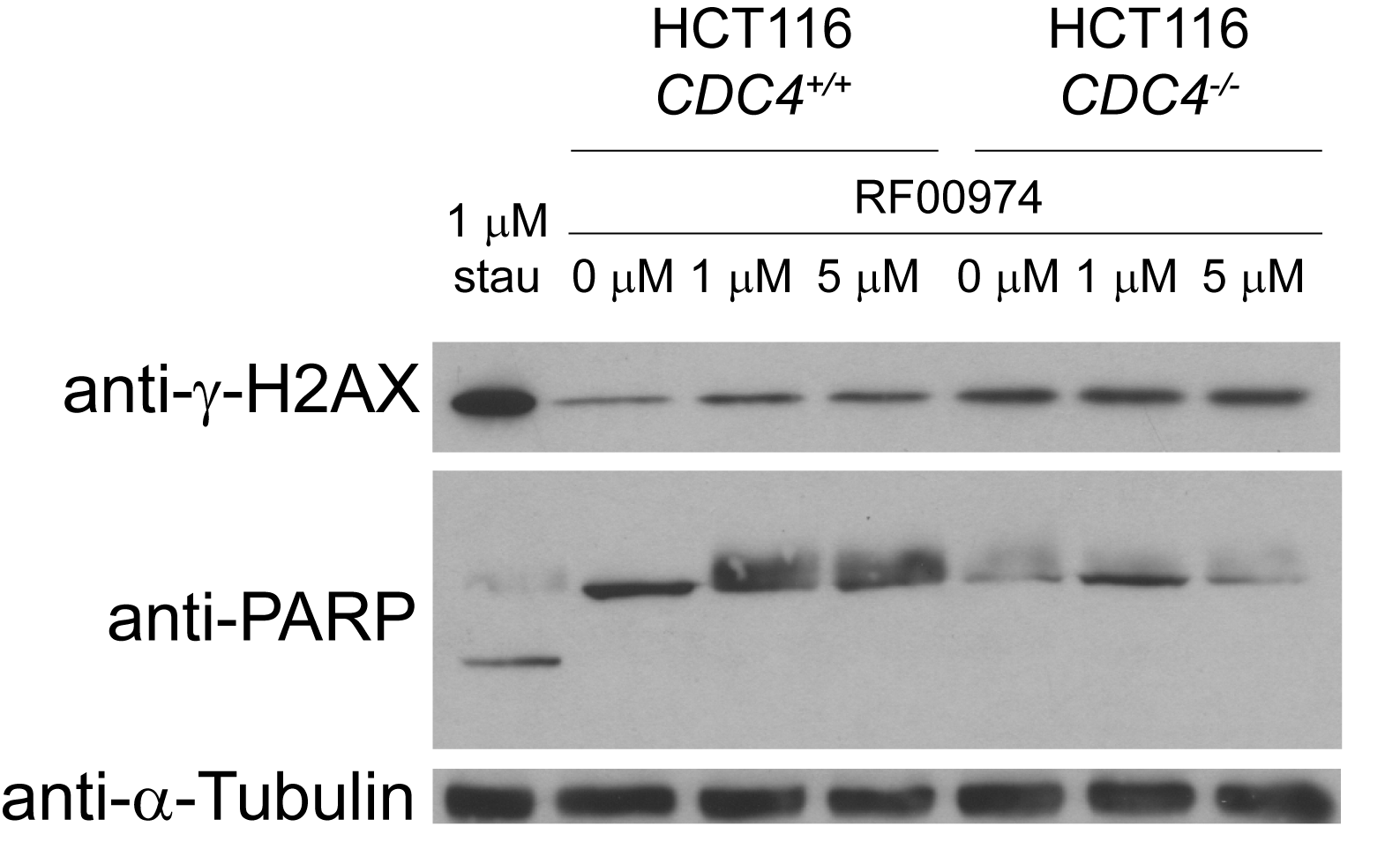

Supplement: Figure S3 — Treatment of cells with RF00974 leads to an increase in phosphorylated H2AX, but not to an increase in apoptosis. Cells were treated with the indicated concentrations of RF00974 for 48 hours before protein was harvested and subjected to Western blot. Staurosporine (Stau) was used as a control to initiate apoptosis. (TIF) [file pgen.1003254.s003.tif]
