# Supplementary material for: An Evolutionarily Conserved Synthetic Lethal Interaction Network Identifies FEN1 as a Broad-Spectrum Target for Anticancer Therapeutic Development
Source: PLoS Genet. 2013 Jan 31;9(1):e1003254. doi: 10.1371/journal.pgen.1003254 (PMC3561056; doi:10.1371/journal.pgen.1003254)
Supplement: Table S1 — siRNA pool silencing in HCT116 cells. Horizontal lines indicate experiments carried out on different days. (DOC) [file pgen.1003254.s004.doc]

**Supplementary Table S1: siRNA pool silencing in HCT116 cells.** Horizontal lines indicate experiments carried out on different days.

Normalized Relative Expected Difference

siRNA N^a^ mean ± SEM^b^ Percent (%)^c^ Percent (%)^d^ (%)^e^

siGAPDH + siGAPDH 32 3173.7 ± 69 100.00 NA NA

siGAPDH + siWDHD1 16 2952.9 ± 150.6 93.00 NA NA

siGAPDH + siFEN1 16 2536.3 ± 89 79.90 NA NA

siGAPDH + siSMC1A 16 2950.1 ± 102.5 93.00 NA NA

siGAPDH + siSMC3 16 3034.1 ± 96.4 95.60 NA NA

siGAPDH + siMRE11A 16 2901.7 ± 99.8 91.40 NA NA

siGAPDH + siCDC4 16 2873.1 ± 128.4 90.50 NA NA

siGAPDH + siBLM 16 2941.3 ± 67.7 92.70 NA NA

siGAPDH + siNIPBL 16 2214.1 ± 98.5 69.80 NA NA

siGAPDH + siSTAG1 16 2320.4 ± 133.8 73.10 NA NA

siWDHD1 + siSMC1A 8 1930.1 ± 103.9 60.80 86.50 29.70

siWDHD1 + siSMC3 8 2131 ± 101.6 67.10 89.00 24.50

siWDHD1 + siMRE11A 8 2195 ± 120.8 69.20 85.10 18.70

siWDHD1 + siCDC4 8 1971.6 ± 60.2 62.10 84.20 26.20

siFEN1 + siSMC1A 8 1663.8 ± 101.3 52.40 74.30 29.40

siFEN1 + siSMC3 8 1491.4 ± 117.6 47.00 76.40 38.50

siFEN1 + siMRE11A 8 1288.8 ± 82.6 40.60 73.10 44.40

siFEN1 + siCDC4 8 1255.5 ± 95.5 39.60 72.30 45.30

siWDHD1 + siBLM 8 1484.9 ± 81.9 46.80 86.20 45.70

siWDHD1 + siNIPBL 8 1144.6 ± 52.6 36.10 64.90 44.40

siWDHD1 + siSTAG1 8 1024.3 ± 83.8 32.30 68.00 52.60

siFEN1 + siBLM 8 1501.8 ± 133 47.30 74.10 36.10

siFEN1 + siNIPBL 8 1271.5 ± 108.7 40.10 55.80 28.10

siFEN1 + siSTAG1 8 1335.3 ± 62.3 42.10 58.40 28.00

siGAPDH + siGAPDH 32 3302.9 ± 85.4 100.00 NA NA

siGAPDH + siWDHD1 16 3146.9 ± 135.2 95.30 NA NA

siGAPDH + siFEN1 16 2601.2 ± 79.6 78.80 NA NA

siGAPDH + siSMC1A 16 3213.8 ± 111.6 97.30 NA NA

siGAPDH + siSMC3 16 4098 ± 142.6 124.10 NA NA

siGAPDH + siMRE11A 16 3850.6 ± 167.4 116.60 NA NA

siGAPDH + siCDC4 16 3315.3 ± 127.4 100.40 NA NA

siGAPDH + siBLM 16 3191.4 ± 105.1 96.60 NA NA

siGAPDH + siNIPBL 16 2538.4 ± 94.7 76.90 NA NA

siGAPDH + siSTAG1 16 2702.6 ± 82.8 81.80 NA NA

siWDHD1 + siSMC1A 8 2599.1 ± 172.9 78.70 92.70 15.10

siWDHD1 + siSMC3 8 3054.4 ± 157 92.50 118.20 21.80

siWDHD1 + siMRE11A 8 2823 ± 179.4 85.50 111.10 23.10

siWDHD1 + siCDC4 8 2137 ± 135.1 64.70 95.60 32.30

siFEN1 + siSMC1A 8 1852.1 ± 83.4 56.10 76.60 26.80

siFEN1 + siSMC3 8 2356.1 ± 108.4 71.30 97.70 27.00

siFEN1 + siMRE11A 8 2302.6 ± 113.5 69.70 91.80 24.10

siFEN1 + siCDC4 8 1512.9 ± 95.9 45.80 79.00 42.10

siWDHD1 + siBLM 8 1891.6 ± 83 57.30 92.10 37.80

siWDHD1 + siNIPBL 8 1494.8 ± 57.1 45.30 73.20 38.20

siWDHD1 + siSTAG1 8 1764.5 ± 73.6 53.40 78.00 31.50

siFEN1 + siBLM 8 2175.3 ± 141.7 65.90 76.10 13.50

siFEN1 + siNIPBL 8 1939.4 ± 75.6 58.70 60.50 3.00

siFEN1 + siSTAG1 8 1483.6 ± 69.7 44.90 64.40 30.30

siGAPDH + siGAPDH 16 2496.9 ± 80.3 100.00 NA NA

siGAPDH + siWDHD1 8 1488.6 ± 73.1 59.60 NA NA

siGAPDH + siFEN1 8 1959.5 ± 90.2 78.50 NA NA

siGAPDH + siSTAG3 16 1954.3 ± 64.5 78.30 NA NA

siGAPDH + siRAD54B 16 1918.6 ± 95.7 76.80 NA NA

siGAPDH + siRNF20 16 1533.6 ± 45.9 61.40 NA NA

siWDHD1 + siSTAG3 8 1062.4 ± 80.3 42.50 46.70 8.80

siWDHD1 + siRAD54B 8 1449.9 ± 144.1 58.10 45.80 -26.80

siWDHD1 + siRNF20 8 663.4 ± 49.6 26.60 36.60 27.40

siFEN1 + siSTAG3 8 1695.3 ± 62.8 67.90 61.40 -10.50

siFEN1 + siRAD54B 8 1225.6 ± 107.1 49.10 60.30 18.60

siFEN1 + siRNF20 8 494.4 ± 37.4 19.80 48.20 58.90

siGAPDH + siGAPDH 16 2978.7 ± 110.1 100.00 NA NA

siGAPDH + siWDHD1 8 2542.1 ± 144.2 85.30 NA NA

siGAPDH + siFEN1 8 1770.8 ± 103.4 59.40 NA NA

siGAPDH + siSTAG3 16 3310.6 ± 102.6 111.10 NA NA

siGAPDH + siRAD54B 16 3442.3 ± 103.8 115.60 NA NA

siGAPDH + siRNF20 16 2096.3 ± 87.5 70.40 NA NA

siWDHD1 + siSTAG3 8 2170.1 ± 52.7 72.90 94.90 23.20

siWDHD1 + siRAD54B 8 1881.6 ± 140.4 63.20 98.60 36.00

siWDHD1 + siRNF20 8 1753.8 ± 93.6 58.90 60.10 2.00

siFEN1 + siSTAG3 8 2164.5 ± 97.5 72.70 66.10 -10.00

siFEN1 + siRAD54B 8 1684.4 ± 104.1 56.50 68.70 17.70

siFEN1 + siRNF20 8 847 ± 54.6 28.40 41.80 32.00

siGAPDH + siGAPDH 24 3474 ± 149.3 100.00 NA NA

siGAPDH + siCHTF8 24 2941.2 ± 126.5 84.70 NA NA

siGAPDH + siSMC1A 8 2941.4 ± 184.3 84.70 NA NA

siGAPDH + siSMC3 8 2947 ± 140.8 84.80 NA NA

siGAPDH + siMRE11A 8 2354.4 ± 103 67.80 NA NA

siGAPDH + siCDC4 8 1990.1 ± 92.6 57.30 NA NA

siGAPDH + siBLM 8 3473 ± 224.8 100.00 NA NA

siGAPDH + siNIPBL 8 2460.7 ± 48.2 70.80 NA NA

siGAPDH + siSTAG1 8 2355.8 ± 68.5 67.80 NA NA

siGAPDH + siSTAG3 8 2284.1 ± 302.7 65.70 NA NA

siGAPDH + siRAD54B 8 3565.7 ± 463.9 102.60 NA NA

siGAPDH + siRNF20 8 1662 ± 219 47.80 NA NA

siCHTF8 + siSMC1A 8 2282.1 ± 69.3 65.70 71.70 8.40

siCHTF8 + siSMC3 8 2692.8 ± 81.5 77.50 71.80 -7.90

siCHTF8 + siMRE11A 8 2869.1 ± 167 82.60 57.40 -43.90

siCHTF8 + siCDC4 8 2115.3 ± 85.4 60.90 48.50 -25.50

siCHTF8 + siBLM 8 1926.1 ± 71.5 55.40 84.60 34.50

siCHTF8 + siNIPBL 8 1301.9 ± 88.4 37.50 60.00 37.50

siCHTF8 + siSTAG1 8 1428.5 ± 68.4 41.10 57.40 28.40

siCHTF8 + siSTAG3 8 2045.5 ± 271.3 58.90 55.70 -5.80

siCHTF8 + siRAD54B 8 2556 ± 330.6 73.60 86.90 15.30

siCHTF8 + siRNF20 8 1759.9 ± 236.6 50.70 40.50 -25.10

siGAPDH + siGAPDH 24 2160.6 ± 104.6 100.00 NA NA

siGAPDH + siCHTF8 24 2120.1 ± 93 98.10 NA NA

siGAPDH + siSMC1A 8 2347 ± 113.8 108.60 NA NA

siGAPDH + siSMC3 8 2031.6 ± 84.3 94.00 NA NA

siGAPDH + siMRE11A 8 2451.8 ± 88.3 113.50 NA NA

siGAPDH + siCDC4 8 1620.8 ± 82.3 75.00 NA NA

siGAPDH + siBLM 8 2566.2 ± 118.5 118.80 NA NA

siGAPDH + siNIPBL 8 1952.8 ± 68.4 90.40 NA NA

siGAPDH + siSTAG1 8 2059.3 ± 51.2 95.30 NA NA

siGAPDH + siSTAG3 8 2665.9 ± 357.5 123.40 NA NA

siGAPDH + siRAD54B 8 2421.9 ± 321.8 112.10 NA NA

siGAPDH + siRNF20 8 2440.1 ± 306.5 112.90 NA NA

siCHTF8 + siSMC1A 8 1984.3 ± 81.5 91.80 106.60 13.80

siCHTF8 + siSMC3 8 1867.9 ± 56.1 86.50 92.30 6.30

siCHTF8 + siMRE11A 8 2348.3 ± 74 108.70 111.40 2.40

siCHTF8 + siCDC4 8 1380.9 ± 72.1 63.90 73.60 13.20

siCHTF8 + siBLM 8 1051.3 ± 74.4 48.70 116.60 58.30

siCHTF8 + siNIPBL 8 1174.9 ± 39.2 54.40 88.70 38.70

siCHTF8 + siSTAG1 8 1889.5 ± 40.1 87.50 93.50 6.50

siCHTF8 + siSTAG3 8 1145.2 ± 146.1 53.00 121.10 56.20

siCHTF8 + siRAD54B 8 1578.7 ± 207.8 73.10 110.00 33.60

siCHTF8 + siRNF20 8 991 ± 128.5 45.90 110.80 58.60

^a^N; number of wells imaged

^b^SEM; standard error about the mean

^c^All values are normalized relative to siGAPDH-silenced controls and shown ± SEM

^d^Calculated by multiplying the normalized relative percentages for the two individual siRNAs

^e^Calculated as: 1 - (Normalized Relative Percent/Expected Percent) × 100. (NA; not applicable)
