# Supplementary material for: An Evolutionarily Conserved Synthetic Lethal Interaction Network Identifies FEN1 as a Broad-Spectrum Target for Anticancer Therapeutic Development
Source: PLoS Genet. 2013 Jan 31;9(1):e1003254. doi: 10.1371/journal.pgen.1003254 (PMC3561056; doi:10.1371/journal.pgen.1003254)
Supplement: Table S2 — siRNA pool silencing in hTERT cells. (DOC) [file pgen.1003254.s005.doc]

**Supplementary Table S2: siRNA pool silencing in hTERT cells.**

Normalized Relative Expected Difference

siRNA N^a^ mean ± SEM^b^ Percent (%)^c^ Percent (%)^d^ (%)^e^

siGAPDH 6 1424 ± 6.467 100.0 ± 1.112 NA NA

siCDC4-pool 6 1257 ± 15.39 88.27 ± 2.646 NA NA

siRAD54B-pool 6 1285 ± 4.835 90.25 ± 8.316 NA NA

siRNF20-pool 6 1343 ± 10.65 94.28 ± 1.831 NA NA

siFEN1-pool 6 1325 ± 8.713 93.01 ± 1.499 NA NA

siCDC4-pool + siFEN1-pool 6 1040 ± 22.24 72.99 ± 3.826 82.10 11.1

siRAD54B-pool + siFEN1-pool 6 975.5 ± 17.93 68.50 ± 3.084 83.94 18.4

siRNF20-pool + siFEN1-pool 6 1049 ± 17.66 73.66 ± 3.038 87.69 16.0

siPLK1-pool 6 22.50 ± 3.631 1.580 ± 0.6245 NA NA

^a^N; number of wells imaged

^b^SEM; standard error about the mean

^c^All values are normalized relative to siGAPDH-silenced controls and shown ± SEM

^d^Calculated by multiplying the normalized relative percentages for siCDC4-pool, siRAD54B-pool or siRNF20-pool with that of siFEN1-pool. (NA; not applicable)

^e^Calculated as; 1 - (Normalized Relative Percent/Expected Percent) × 100. (NA; not applicable)
