# Supplementary material for: An Evolutionarily Conserved Synthetic Lethal Interaction Network Identifies FEN1 as a Broad-Spectrum Target for Anticancer Therapeutic Development
Source: PLoS Genet. 2013 Jan 31;9(1):e1003254. doi: 10.1371/journal.pgen.1003254 (PMC3561056; doi:10.1371/journal.pgen.1003254)
Supplement: Table S3 — Synthetic Lethality between FEN1 and cancer genes in hTERT cells. Horizontal lines indicate experiments carried out on different days. (DOC) [file pgen.1003254.s006.doc]

**Supplementary Table S3: Synthetic Lethality between FEN1 and cancer genes in hTERT cells.** Horizontal lines indicate experiments carried out on different days.

Normalized Relative Expected Difference

siRNA N^a^ mean ± SEM^b^ Percent (%)^c^ Percent (%)^d^ (%)^e^

siGAPDH 6 1412 ± 7.073 100.0 ± 1.227 NA NA

siCDC4-1 6 1325 ± 20.39 93.81 ± 3.538 NA NA

siCDC4-3 6 1366 ± 16.01 96.78 ± 2.354 NA NA

siFEN1-2 6 1369 ± 11.06 96.99 ± 1.919 NA NA

siCDC4-1 + siFEN1-2 6 1003 ± 13.48 71.05 ± 2.339 90.99 21.9

siCDC4-3 + siFEN1-2 6 867 ± 15.39 61.41 ± 2.671 93.87 34.6

siPLK1 6 25 ± 5.961 1.771 ±1.034 NA NA

siGAPDH 6 1462 ± 14.29 100.0 ± 2.394 NA NA

siRAD54B-1 6 1380 ± 14.53 94.35 ± 2.433 NA NA

siRAD54B-2 6 1383 ± 9.731 94.59 ± 1.630 NA NA

siFEN1-2 6 1389 ± 10.46 94.95 ± 1.752 NA NA

siRAD54B-1 + siFEN1-2 6 1071 ± 19.24 73.22 ± 3.223 89.59 18.3

siRAD54B-2 + siFEN1-2 6 1114 ± 21.09 76.19 ± 3.532 89.81 15.2

siPLK1 6 54.5 ± 10.52 3.73 ± 1.761 NA NA

siGAPDH 6 2119 ± 24.73 100.0 ± 2.860 NA NA

siRNF20-1 6 1901 ± 23.46 89.71 ± 2.713 NA NA

siRNF20-3 6 2036 ± 16.68 96.12 ± 1.928 NA NA

siFEN1-2 6 1914 ± 24.25 90.33 ± 2.804 NA NA

siRNF20-1 + siFEN1-2 6 1359 ± 20.76 64.16 ± 2.400 81.04 20.8

siRNF20-3 + siFEN1-2 6 1259 ± 30.25 59.41 ± 3.497 86.83 31.6

siPLK1 6 96 ± 19.68 4.539 ± 2.276 NA NA

^a^N; number of wells imaged

^b^SEM; standard error about the mean

^c^All values are normalized relative to siGAPDH-silenced controls and shown ± SEM

^d^Determined by multiplying the normalized relative percentages for siCDC4-1 or siCDC4-3 with that of siFEN1-2. (NA; not applicable)

^e^Calculated as; 1 - (Normalized Relative Percent/Expected Percent) × 100. (NA; not applicable)
