# Supplementary material for: An Evolutionarily Conserved Synthetic Lethal Interaction Network Identifies FEN1 as a Broad-Spectrum Target for Anticancer Therapeutic Development
Source: PLoS Genet. 2013 Jan 31;9(1):e1003254. doi: 10.1371/journal.pgen.1003254 (PMC3561056; doi:10.1371/journal.pgen.1003254)
Supplement: Table S4 — Antibodies employed in Western blots in this study. (DOC) [file pgen.1003254.s007.doc]

**Supplementary Table S4: Antibodies employed in Western blots in this study.**

| **Protein target** | **Supplier** | **Catalog number** | **Dilution** |
| --- | --- | --- | --- |
| CDC4 | Abcam | ab12292 | 1:1000 |
| FEN1 | Abcam | ab462 | 1:1000 |
| FEN1 | Abcam | ab17993 | 1:3000 |
| γ-H2AX | Millipore | 05-636 | 1:2000 |
| MRE11A | Abcam | ab397 | 1:10 000 |
| PARP | BD | 556494 | 1:500 |
| RAD54B | Abcam | ab83311 | 1:1000 |
| RNF20 | Abcam | ab32629 | 1:3000 |
| SMC1A | Abcam | ab9262 | 1:1000 |
| SMC3 | Abcam | ab9263 | 1:1000 |
| WDHD1 | Sigma | HPA001122 | 1:1000 |
| Tubulin, alpha | Abcam | ab7291 | 1:10 000 |
| Tubulin, alpha | Abcam | ab18251 | 1:10 000 |
| Mouse IgG (HRP-conjugated) | Jackson ImmunoResearch | 111-035-144 | 1:10 000 |
| Rabbit IgG (HRP-conjugated) | Jackson ImmunoResearch | 111-035-146 | 1:10 000 |
